# Medical Colleges

**Published:** 1877-10

**Authors:** 


Medical Colleges.—Notwithstanding the very great stringency in financial matters, medical colleges seem to be opening with larger than the usual number of students.

The extension of the term, as favored by the Association of Colleges, does not, at any rate, interfere with their patronage The classes, so far as we have noticed reported, seem to be larger than heretofore, when the opening occurred in November. This speaks well for those preparing to become members of the medical profession and for their preceptors, who advise that course of study which gives the greatest facilities for thorough preparation. While we do not favor the idea of time of study being made a test of preparation to be relied on in granting the degree, we are conscious of the fact that three months do not allow sufficient time to teach, fully, any branch in the curriculum of studies. It is gratifying to know that when we have served our time in the profession, our mantle will fall upon those who seek something more than the empty title of M.D.

The Louisville, Nashville, and Atlanta colleges have larger classes than last year. Some of them, and perhaps all, commence the present course with a larger number than at any time since the war.

The Atlanta Medical College has in attendance, during the month of October, more students than are usually present in November, and a few days more will doubtless swell the number beyond that of any class since the war.

Much interest is manifested in the various departments of study, and a seeming determination to comprehend and retain the fundamental principles of medicine.

